# A survey on the implementation of environmental health monitoring in mouse facilities in German-speaking countries

**DOI:** 10.1371/journal.pone.0334442

**Published:** 2025-10-23

**Authors:** Esther Mahabir, Katja Schmidt, Thomas Kolbe, Stephanie Buchheister, Manuel Miller

**Affiliations:** 1 Comparative Medicine, Center for Molecular Medicine Cologne (CMMC), Faculty of Medicine and University Hospital Cologne, Cologne, Germany; 2 Microbiological Diagnostics, German Cancer Research Center (DKFZ), Heidelberg, Germany; 3 Institute of In vivo and In vitro Models, University of Veterinary Medicine Vienna, Vienna, Austria; 4 Department of Agricultural Sciences, University of Natural Resources and Life Sciences, Vienna, Austria; 5 Institute for Laboratory Animal Science and Central Animal Facility, Hannover Medical School, Hannover, Germany; 6 Core Facility Laboratory Animal Services, Helmholtz Munich, Neuherberg, Germany; Anhui University of Chinese Medicine, CHINA

## Abstract

The health status of laboratory animals plays a decisive role not only for the health and welfare of the animals but also for the validity of study results. In recent years, there has been an increasing number of publications on environmental health monitoring (EHM), which uses molecular biological methods to detect nucleic acids of infectious agents in individually ventilated cage systems, e.g. in exhaust air dust. This monitoring strategy can reduce the number of mice used for health monitoring in conformity with the 3Rs. Numerous studies have shown that EHM is reliable and sensitive and is, therefore, a useful method for health monitoring of mice. An online survey was created to assess the prevalence of the use of EHM in Germany, Austria, and Switzerland and to better understand the factors influencing its use in animal facilities. The survey revealed that the majority of facilities primarily equipped with individually ventilated cage systems already use EHM to varying degrees, replacing between 8 and 1200 animals per year and facility. However, the predominant strategy is still the use of (sentinel) animals for health monitoring. Beliefs on factors such as cost, reliability and the number of false-positive results differ significantly between facilities that predominantly use either animals or EHM. Additionally, the choice of monitoring strategy may be influenced by the existing cage system and the availability of a decontamination option for the equipment. The evaluation of the survey showed that there is still a gap in knowledge and a demand for specific training on the topic of health monitoring and especially on EHM.

## Introduction

Health monitoring (HM) of laboratory mice is an inherent task in animal research facilities and aims to ensure the health and welfare of the animals and personnel as well as the reproducibility and validity of experimental results. Historically, routine HM of mice has been performed via sentinel mice, using primarily soiled-bedding sentinels (SBS). SBS mice are exposed to pooled soiled bedding from colony cages at the time of cage changing over a period of 3–6 months. Then, they are usually killed, necropsied, and used for diagnostics. In Europe, the Federation of European Laboratory Animal Science Associations (FELASA) published recommendations for the HM of mice, according to which HM for relevant infectious agents is performed. The presence or absence of these agents is monitored either quarterly or annually, depending on their prevalence and importance for biomedical research [1]. However, there are many reports on the lack of or inefficient transmission of infectious agents via soiled bedding to sentinel mice [2–11].

Environmental Health Monitoring (EHM) is a relatively recent development that offers a more effective method of HM and avoids the need for animal testing. EHM refers to the surveillance of infectious agents in mouse colonies without the use of live animals, whereby samples are assayed via conventional polymerase chain reaction (PCR) or quantitative (q)PCR. The sample types suitable for EHM strongly depend on the type of cages and racks used in the monitored housing unit. The exhaust air may be unfiltered or filtered at the cage level. In case of unfiltered exhaust air at the cage level, dust samples are usually collected from the plenum or air handling units using swabs or special matrices [4,6,11–21]. This is termed exhaust dust testing (EDT) according to a recent suggestion [22]. Samples for EHM can also be taken from any type of cage and IVC rack system even if the exhaust air is filtered at the cage level. One method for routine HM replacing live animals is sentinel-free soiled bedding (SFSB) testing [23], which was developed after EDT [21,24–27]. With this method, collection and pooling of soiled bedding is done in the same way as for HM with SBS but without animals in the cage. Filter material, sterile swabs or commercially available dedicated matrices for the specific rack type are used to collect the nucleic acid-containing material. HM results via SFSB were reported to be equal or superior to those using SBS [21,24–26,28–30]. Also, in IVCs equipped with a filter at the cage level, PCR analysis of the filters was useful in detecting various infectious agents [28]. SFSB has proven to be an appropriate and reliable testing method during quarantine [31]. In addition, samples can also be taken from other surfaces of the room and equipment and then used for PCR analysis [32,33].

In the last two decades, multiple studies showed that EHM was effective in detecting various infectious agents when used exclusively or as an adjunct method to traditional HM strategies [4,5,7,8,11–18,20,21,24,25,28,29,34–40]. Furthermore, a systematic review of 42 peer-reviewed publications, comparing the use of EHM versus SBS, showed a higher detection rate of infectious agents in environmental samples [22]. Notably, in that review, EDT and SFSB failed to detect a particular pathogen only in six and one case, respectively, while SBS failed in 21 cases. In addition to improving the diagnostic success, the implementation of EHM in animal facilities allows for a reduction in the number of mice used for routine HM, thereby contributing to the 3Rs. Furthermore, EHM can lead to a reduction in labour and costs incurred [23,41].

However, many institutions have not yet adopted EHM as the sole or as an adjunct method of HM and, despite the advantages of EHM, still use SBS. To clarify this discrepancy and to elucidate the underlying reasons, Luchins and colleagues [23] conducted a survey in 2021 and collected data from animal facilities on the use of EHM. However, mainly the situation in North America was reported (89% of participants) as only 17 of the 158 participants (10%) from 111 institutions were located in Europe [23]. The results showed that in 2021, only 11% of the institutions used EHM as the sole HM strategy and 87% of surveyed institutions still used SBS either alone (41%) or as a combination of both methods (46%). Notably, the use of EHM either solely or as an adjunct method in these surveyed institutions would have led to a reduction of over 20,000 rodents annually [23].

To specifically assess the prevalence of the use of EHM in laboratory mouse facilities in Germany, Austria, and Switzerland and to determine the individual factors influencing its use, the Committee for Hygiene of the Society for Laboratory Animal Science (GV-SOLAS) conducted a very similar survey. The aim of this survey was to systematically assess the current state of knowledge about EHM, its actual implementation in institutions, and potential barriers to its adoption. Likewise, we also aimed to identify supporting factors for its increased use in institutions and the reduction of the number of mice used for routine HM, thereby contributing to the reliability of HM results and animal welfare.

## Materials and methods

### Measures and content

A questionnaire in German language was developed by members of the Committee for Hygiene of the GV-SOLAS (Gesellschaft für Versuchstierkunde - Society of Laboratory Animal Science) who are experts in the field of HM and/or heads of rodent animal facilities. This committee develops white papers on HM and actively contributes to continuing education, particularly through workshops at the laboratory animal science conferences. Prior to the development of the survey, which was generated using “LimeSurvey” (Community Edition, Version 5.6.68 + 240625), a thorough review of the literature was performed. The questions in the survey were based on identified gaps in the implementation of EHM in German-speaking countries, as recognized by the GV-SOLAS, and on the EHM survey by Luchins et al. [23]. The survey was sub-divided into five sections containing 33 questions and a comment field (see supporting information in S1 Table, which was translated into English). It focused on 1) general information about the institution and its caging systems, 2) the level of knowledge about the different HM strategies and sources of information, 3) the predominant HM strategy, sampling methods and diagnostics used, 4) the availability of decontamination systems, 5) current and potential reduction of animals via EHM, the actual knowledge and the perception of differences between the use of SBS and EHM, particularly with respect to factors influencing the choice of HM strategy as well as the future probability for implementing EHM, including the acceptance of health reports and the influence of further training or a FELASA recommendation on adopting EHM.

In all 33 questions, several options for answers were given (see S1 Table); 26 questions allowed single-answers and 5 questions multiple answers. Questions 18 and 19, which relate to the reduction of animal use, as well as the comment field 34 were open-ended text fields and voluntary. All other questions were mandatory to complete. Thresholds used in the questionnaire divided the categories into meaningful units: Question 4 (number of cages) indicated small, medium and large facilities. The term “predominantly” is understood as markedly over 50% (questions 12 and 14). The threshold of cage equipment with at least 50% IVCs for individual evaluations was chosen because, in Europe, the use of EHM for routine HM is primarily implemented in IVC systems (EDT), while the use of SFSB has played a subordinate role so far.

### Participants, timeline, and procedures

Invitation emails to participate in the survey were sent exclusively to the animal facility managers, with instruction to complete only one questionnaire per institution/facility. Therefore, the number of participants and the number of institutions are treated as equivalent in this manuscript. The online survey was designed to be completed either by the animal facility managers themselves or by the person responsible for health monitoring, ensuring familiarity with their institution’s HM program. The link to the survey was distributed via an email distribution list on May 15th, 2024, followed by a reminder two weeks later, and remained open until June 7th, 2024. An estimated duration of 15 minutes was calculated for completing the questionnaire. It was possible to interrupt the completion of the questionnaire at any time and to save the answers.

### Data analysis

The responses were categorized based on the plausibility of answers given: 1) all answers that were fully consistent were included in the evaluation, 2) one single answer was contradictory to all other answers and therefore, only this answer was excluded from the evaluation, 3) more than one answer was contradictory and it was not possible to assess the overall HM strategy of the facility. In the latter case, the complete questionnaire was excluded from further analysis. Each dataset was independently reviewed by two co-authors. In case of disagreement, a third co-author was consulted for evaluation. Subsequently, all five co-authors jointly evaluated the categories. The original questionnaire differentiated between IVC cages with and IVC cages without cage-level air filtration (questions 5b and 5c as well as 10 and 11). However, answers given in this context were often contradictory, which is why we reduced this bias by combining both IVC types (with and without cage-level air filtration) under “IVC systems” without further differentiation. Therefore, answers for questions 5b and 5c (husbandry system and percentage of IVCs) and answers for questions 10 and 11 (IVCs and EHM) were merged, respectively. The results for each question were summarized descriptively. In the figures, “n” indicates the number of questionnaires that were included in the evaluation of the specific question (category 1 questionnaires plus category 2 questionnaires minus the respective single responses that were excluded). For better understanding and comparison of data, results are presented generally as percentages with the exception of data for the number of countries and the number of participants in each category of responses. In each figure legend, we included the numbers to which the percentages refer. Participants who indicate “low” or “very low” knowledge of EHM (questions 6–8) were not excluded from the analysis. In particular, questions 20–25 aim to reveal biases. The answers to these questions reflect personal beliefs and not objective data, especially since participants with low or very low knowledge of EHM or those who have not yet implemented EHM lack personal experience. The data were exported and analysed using Microsoft Excel. Graphs were created with GraphPad Prism (10.4.1).

### Ethics statement

For this survey, no animal experiments were conducted, and no patient information or samples were analysed. Participants were informed about the purpose, content, duration, and voluntary nature of the survey in the invitation email of the survey and that the anonymised results would be published. Informed consent was obtained online through participation and submission of responses via the survey tool. As the survey was completely anonymous, it was not possible to obtain written or verbal consent, which ensured that participants could not be traced at any time point. Participation was voluntary and restricted to individuals over 18 years of age. No personal or sensitive data were collected. Ethical approval was not required for this survey since it did not involve any form of intervention, sensitive data, vulnerable populations or minors, thereby absolutely excluding any harm to the participants.

## Results

### Demographic data and categorization of answers

In total, 99 questionnaires were fully completed, 80 of which were from Germany, 10 from Austria, and 9 from Switzerland. Based on the conclusiveness of answers, 73 questionnaires were assigned to category 1 (all answers were fully consistent) and 18 questionnaires to category 2 (one single answer was contradictory to all other answers and, therefore, only this answer was excluded from the evaluation). Eight questionnaires were assigned to category 3 (more than one answer was contradictory and it was not possible to assess the overall HM strategy of the facility) and were thus excluded from the evaluation. Therefore, a total of 91 questionnaires, representing 91 facilities, were included in the evaluation of this survey (see Table 1). Most of the facilities (84%) belonged to the academic sector and only 16% to the industry sector. The questionnaires of categories 1 and 2 were filled out primarily by facility managers (n = 59), veterinarians (n = 48) or biologists (n = 8). Seven participants indicated that they had a different professional background indicated by „other“. Notably, some participants had more than one academic background and role.

**Table 1:** Demographics and information about animal facilities and participants from questionnaires included in the data analysis (n = 91).

Parameter	n	% of total
**Location**		
Germany	74	81
Austria	8	9
Switzerland	9	10
**Institution Type**		
Academic	76	84
Industry	15	16
**Role**		
Manager	29	32
Veterinarian	25	27
Manager/Veterinarian*	22	24
Veterinarian/Biologist*	1	1
Manager/Biologist*	6	7
Manager/other*	2	2
Biologist	1	1
Other	5	5

*A combination of answers was possible.

### Caging systems

Approximately half of the facilities maintained between 1,000 and 10,000 cages (49%), followed by large facilities with more than 10,000 cages (36%) and very small animal facilities with less than 1,000 cages (14%). The responses showed that, in some cases, a combination of different cage systems was used in the same animal facility. Most facilities had IVCs (90%) and/or open cages (49%), and 19% indicated other cage systems that were not further specified.

### Health monitoring strategy

**Fig 1:**
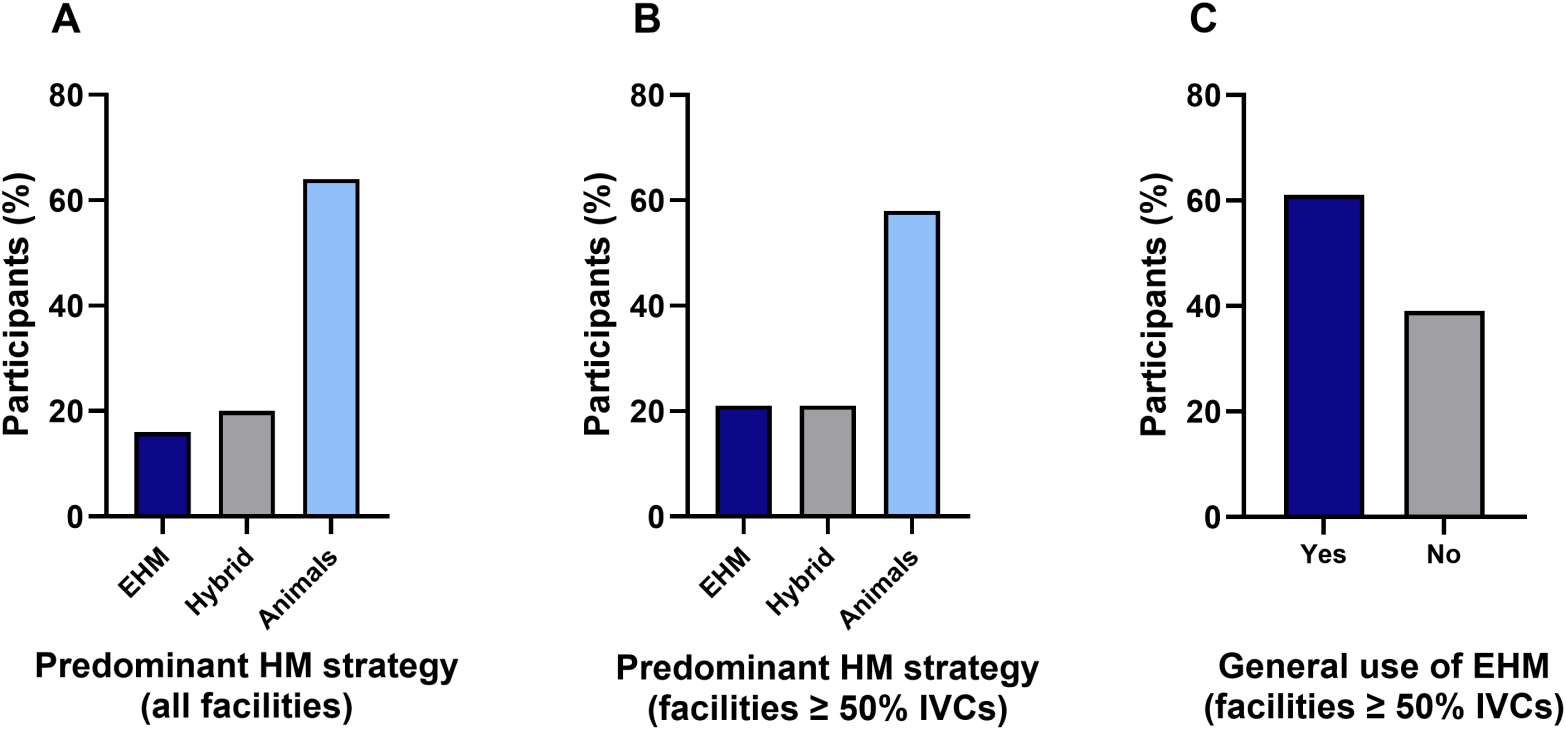
Predominant health monitoring (HM) strategy currently used by the participating institutions. “EHM” = predominant use of environmental samples, “Hybrid” = use of a combination of environmental samples and animals, “Animals” = predominant use of animals. A) Predominant HM strategy in all facilities (n = 91) regardless of the cage type used. B) Predominant HM strategy in facilities equipped with at least 50% IVCs (n = 71). C) General use of EHM in facilities equipped with at least 50% IVCs regardless of the degree to which it is used in the three different HM programs. Here, EHM may be applied in various ways: as a supplement to animal testing (e.g., via swabs), as part of a hybrid system or as the predominant health monitoring strategy (n = 61).

In German-speaking countries, the predominant HM strategy is still based on animals (64%), followed by a combination of animals and EHM (“Hybrid”; 20%) and predominantly EHM (16%) (Fig 1A). Notably, when only animal facilities equipped with at least 50% IVC cages were considered, the results were similar (Fig 1B). They either used predominantly animals (58%), a combination of animals and EHM (“Hybrid”, 21%) or predominantly EHM (21%). In some of the facilities that predominantly used animals for HM, the strategy was supplemented with EHM samples, including environmental swabs (Fig 1B). The majority of these participants (with ≥ 50% IVCs in their animal facilities) stated that they already used EHM as part of their HM program (59%, Fig 1C). Most participants (92%) submitted their samples to external commercial diagnostic laboratories, while only 8% of the facilities had access to in-house diagnostic laboratories. None of the latter participants exclusively used EHM.

### Self-assessment

**Fig 2:**
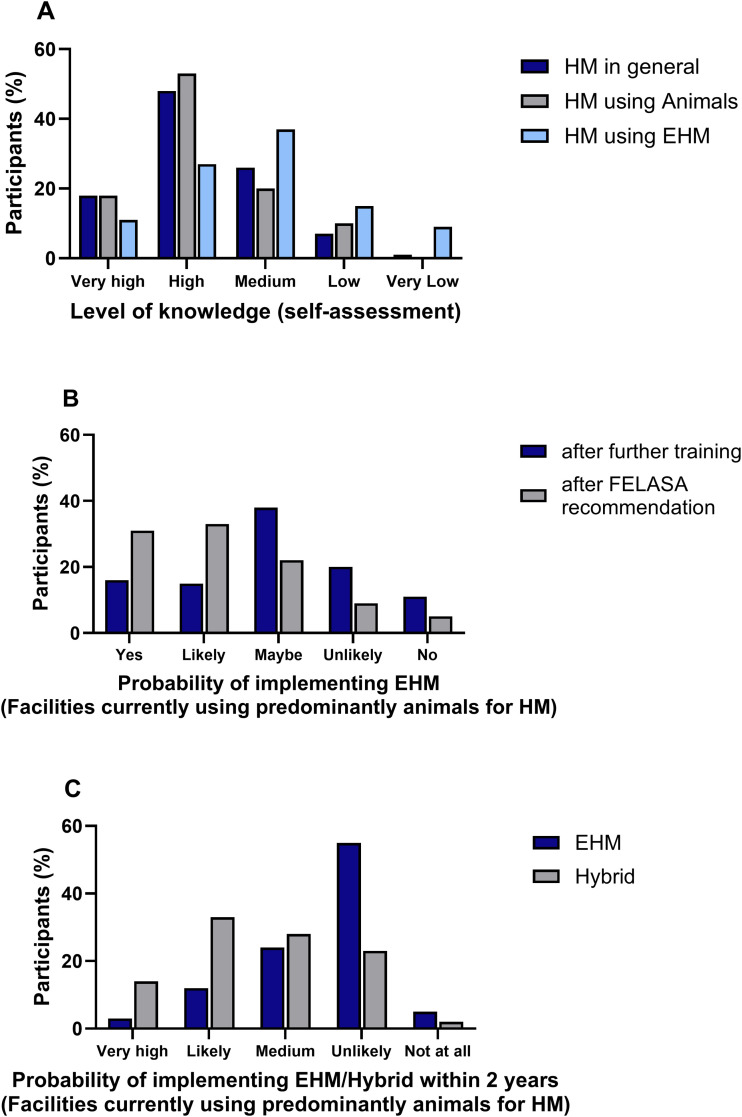
Self-assessment and future implementation of EHM. A) Level of knowledge about HM overall using animals or environmental samples (n = 91). B) Likelihood of implementing EHM (n = 55). C) Likelihood of implementing either EHM or a hybrid system within the next two years (n = 58).

The self-assessment questions reflected only the participants’ personal evaluation. Even if the reported level of knowledge was low, the participants were still expected to answer the other questions accurately. Data related to the self-assessed level of knowledge within the facility regarding HM in general, the use of animals, and EHM are shown in Fig 2A. Most participants (90%) rated the level of knowledge about HM based on animals as “high” followed by “medium”, and “very high”, only 10% of the participants reported this factor as “low”. The majority (76%) rated their knowledge about EHM as “medium” followed by “high”, and “very high”. A total of 24% of the participants classified their level of knowledge about EHM as “low” (15%) or even “very low” (9%). Focusing on the group that predominantly used animals for testing, the majority (38%) was undecided as to whether they would be more willing to implement EHM after specific training on this topic and selected “maybe”. This is also reflected in the fact that 31% of participants chose either “yes” or “likely” while 31% chose “unlikely” or “no” (Fig 2B). The most decisive factor for participants seemed to be whether the methodology would be published in a future FELASA recommendation. If this would be the case, 64% of participants answered either “yes” (31%) or “likely” (33%) when asked whether they would then be willing to implement EHM. When the group that predominantly used animals was asked about plans to introduce EHM or a hybrid method within the next two years, they were more inclined to consider using a hybrid system rather than exclusively adopting EHM (Fig 2C). The main resources used to obtain information and further training on the topic of HM in general by participants were conference attendance (89%) followed by literature (76%), commercial diagnostic laboratories (75%) and discussions with colleagues (71%).

### Factors influencing the HM strategy

**Fig 3:**
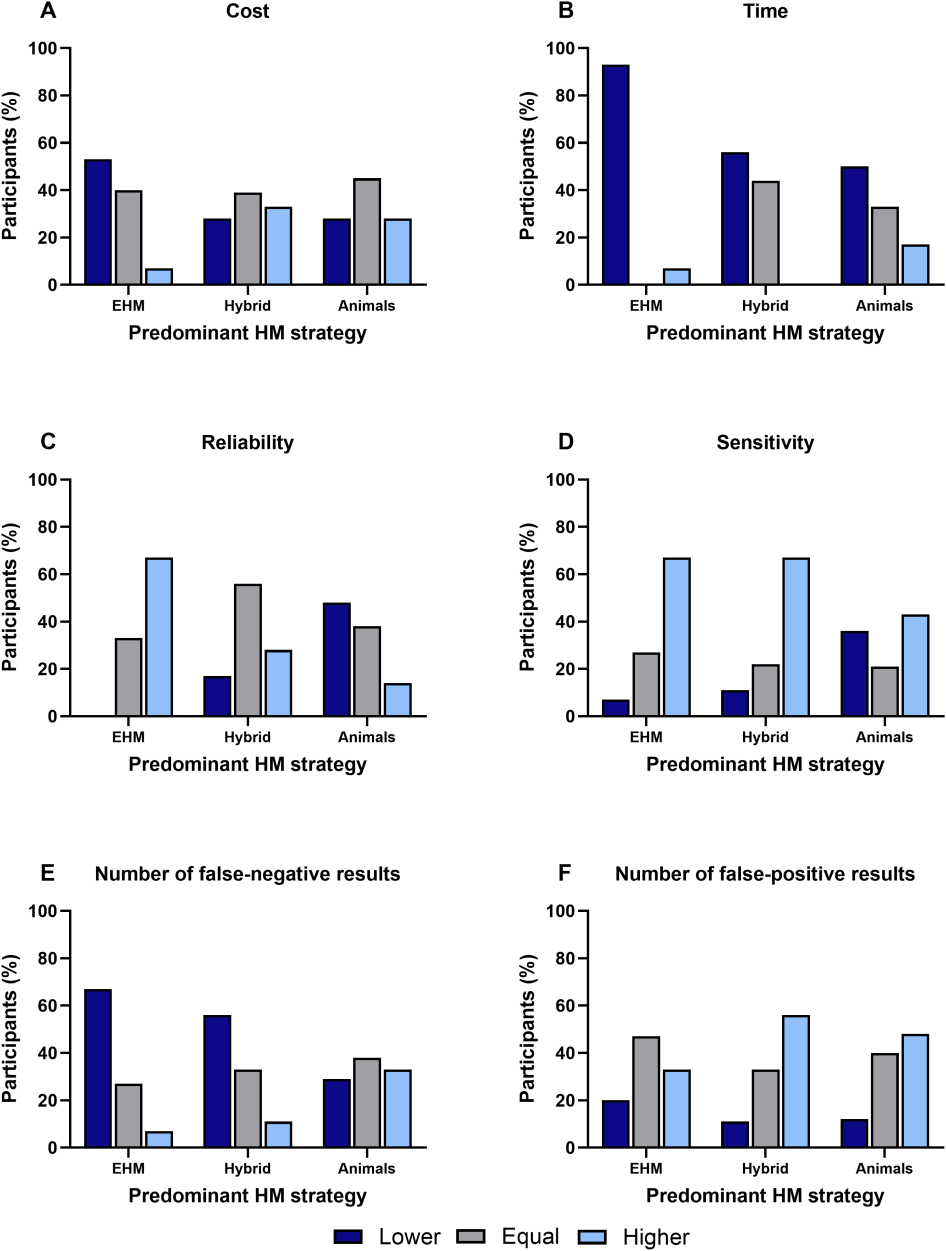
Factors influencing the future choice of the HM strategy according to the current predominant HM strategy. Participants were asked to compare (lower, equal, higher) the use of environmental health monitoring (EHM) to the use of animals (sentinels) regarding A) cost, B) time, C) reliability, D) sensitivity, E) number of false-negative results, and F) number of false-positive results. “EHM” = predominant use of environmental samples (n = 15), “Hybrid” = use of a combination of environmental samples and animals (n = 18), “Animals” = predominant use of animals (n = 58).

The response concerning factors such as cost, time, reliability, sensitivity, false-negative or false-positive results, which currently influence or may influence the decision to use a specific HM strategy and possible beliefs against the use of EHM, are shown in Fig 3.

Most “EHM” participants (53%) reported cost reductions by switching to the analysis of environmental samples. When animals were used in a combination or predominantly, the perception that the costs were or would be equivalent slightly prevailed (“Hybrid” facilities: 39%, “Animal” facilities: 45%) (Fig 3A). All groups agreed that the use of EHM could save time compared to the use of animals. This is most evident in the “EHM” group, where 92% reported that time could be saved by switching to the new methodology. Also, in the other facilities, the opinion prevailed that time savings can be achieved by using EHM (“Hybrid” facilities: 53%, “Animal” facilities: 50%) (Fig 3B). When asked whether the reliability of HM was or would be increased through EHM, the results varied considerably between the groups. In the “EHM” group, no participant reported a decrease in reliability, and the majority (67%) stated an improvement. In the “Hybrid” group, the reliability was primarily assessed as “equal” (56%). The “Animals” group assessed the reliability of using EHM mostly as “lower” (48%) (Fig 3C). Across all groups, EHM was perceived as having a greater sensitivity than traditional animals-based HM. Notably, the sensitivity ratings were similar between participant employing EHM exclusively and those using a Hybrid approach (Fig 3D). The majority of participants in the “EHM” and “Hybrid” groups reported that false-negative results occurred less frequently (67% and 56%, respectively) when testing environmental samples. In the “Animals” group, the answers were more evenly distributed (Fig 3E). The number of false-positive results was assessed to be “higher” in the “Hybrid” group (56%) and in the “Animals” group (48%). False-positive results in EHM, assessed as “equal”, occurred with similar frequency in facilities that predominantly used EHM and those that primarily relied on animals for HM (47%) (Fig 3F).

### Use of EHM in accordance with the 3Rs

Thirty participants who already used EHM provided more detailed information about the current animal savings by using environmental samples as a replacement or as a supplement to the use of animals. A reduction with a wide range between 8 and 1,200 animals per facility and year was reported. In total, 6,331 animals per year were already replaced in these animal facilities. A further 32 participants predicted potential savings ranging from 4 to 2,500 animals per facility and year if they would switch to EHM or increase the use of EHM, resulting in a replacement of further 6,517 animals per year.

### Decontamination of racks

**Fig 4:**
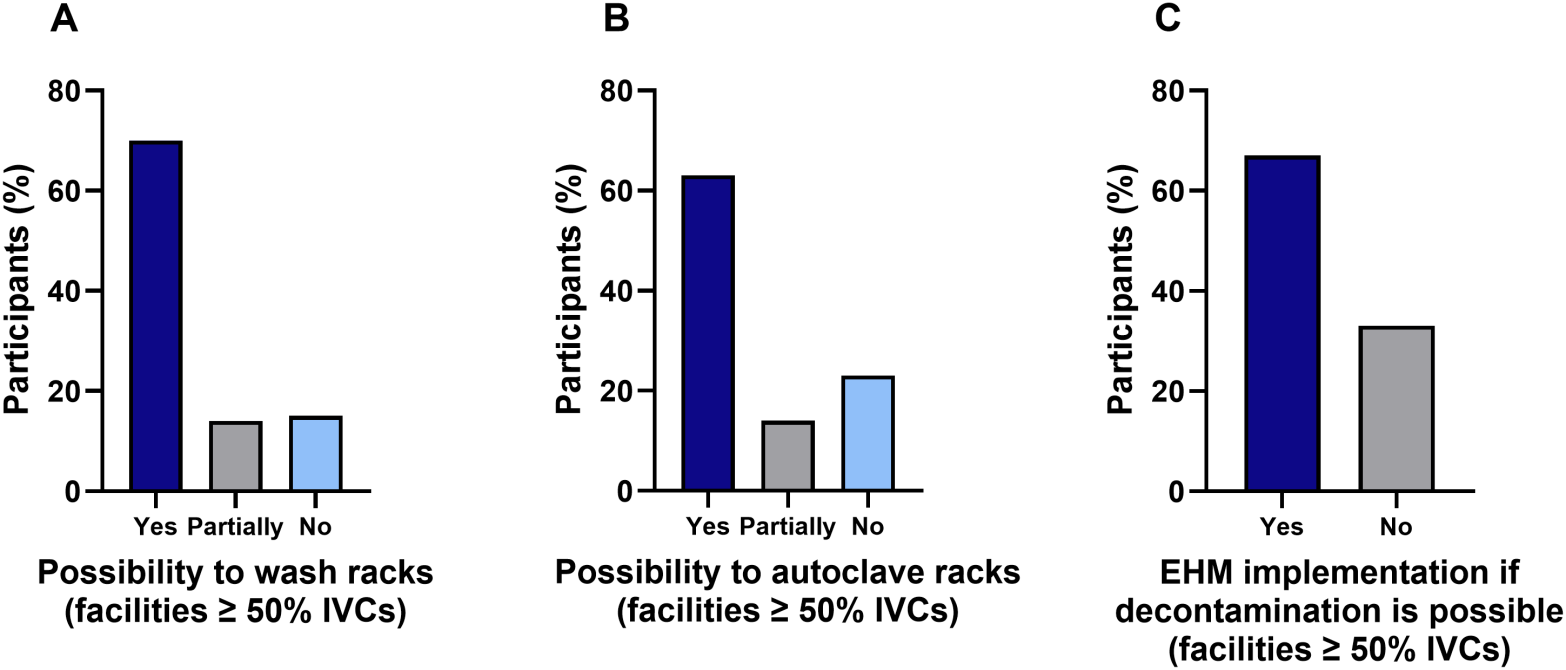
Availability of decontamination options for IVC racks in animal facilities. A) Possibility to wash IVC racks in facilities equipped with at least 50% IVCs (n = 71). B) Possibility to autoclave IVC racks in facilities equipped with at least 50% IVCs (n = 71). C) Assessment of the facilities that currently have no decontamination option as to whether they would use EHM if a decontamination option were available (n = 15).

The majority of facilities equipped with at least 50% IVCs (70%) stated that they have the possibility to wash racks. Nearly 1/3 of participants had only partial (14%) or no option (16%) to wash their racks (e.g. in a rack washer) (Fig 4A). Similarly, just over 1/3 of participants had only partial (14%) or no option (23%) to autoclave their racks (Fig 4B). Of those who could not decontaminate racks and did not use EHM, 2/3 of participants indicated that they would implement EHM if appropriate decontamination equipment was available in their facilities (Fig 4C).

### Acceptance of health certificates

**Fig 5:**
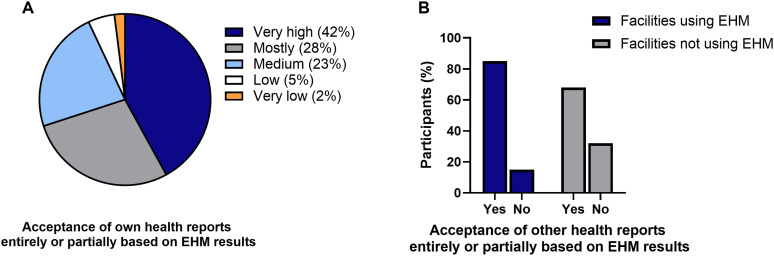
Acceptance of health certificates. A) Assessment of the acceptance rate of EHM-based health certificates by recipient facilities (n = 43). B) Information of participants on the acceptance of health reports from other facilities that were based entirely or partially on EHM results (n = 41 for “facilities using EHM“, n = 37 for “facilities not using EHM“).

Our survey data revealed that there was a high rate of acceptance of the exporting facility’s health certificates, particularly when they were based entirely or partially on EHM results (Fig 5A). Notably, the overwhelming majority of participants (93%) reported “medium”, “high” or “very high” acceptance of their own health certificates when exporting mice, while only 5% and 2% of the participants reported “low” or a “very low” acceptance, respectively (Fig 5A). Furthermore, most participants accepted health certificates based entirely or partially on EHM results, particularly when their own facility also employed EHM (86% vs. 68%) (Fig 5B).

## Discussion

This survey evaluates the prevalence of EHM in animal facilities in German-speaking countries and the factors influencing its implementation. Questionnaires from 99 institutions in Germany, Austria, and Switzerland were submitted, of which 91 questionnaires were included in the evaluation of the survey. The main findings provide information to better understand the challenges associated with the implementation of and/or the transition to EHM. In 2021, Luchins and colleagues [23] published a similar survey, in which 111 institutions primarily from the United States of America participated. They identified several factors that promote the use of EHM as well as various barriers to the implementation of this method. In Europe, over the last two decades, many animal facilities housing laboratory rodents switched their caging systems from open cages to IVCs. However, housing laboratory rodents in IVCs poses a challenge for HM when using soiled-bedding sentinels or colony animals since the cages are designed to prevent the spread of microorganisms, including pathogens. Therefore, each cage represents a single hygienic unit, which reduces the prevalence and complicates the detection of pathogens within the animal husbandry.

Our survey showed that the use of SBS is still widespread in German-speaking countries, with 64% of participants relying on it as the predominant health monitoring strategy and 20% using a hybrid method. These results are different to those reported by Luchins et al. 2021 survey [23] where 41% of the participants used SBS only and 46% implemented a hybrid method. Their data indicate that more animal facilities in the latter study than in the German-speaking countries have made the transition to implementing EHM. Despite advancements in monitoring methods, 84% of animal facilities in our study and 87% reported by Luchins et al. [23] still use SBS as part of their routine HM strategy.

SBS have several limitations since their effectiveness depends on multiple factors including the selection of the sentinel animals (age, strain, immune and health status) [43–47], pathogen prevalence, environmental survival, transmission mode and shedding duration. Some pathogens are poorly or not transmitted via soiled bedding (e.g. lymphocytic choriomeningitis virus, Sendai virus, pneumonia virus, adenoviruses, polyomaviruses, mites and *Pneumocystis murina*) or survive only briefly outside the host (*Rodentibacter* spp., *Streptobacillus moniliformis*, *Mycoplamsa* spp., *Klebsiella* spp., *Pseudomonas* spp., *Helicobacter* spp.) [3,40,48,49]. Consequently, relying solely on SBS testing is inadequate for detecting a broad range of pathogens, particularly those with low prevalence or limited environmental stability such as *Helicobacter* spp. [8,11,29,50], *Rodentibacter* spp. [4,6], *Staphylococcus aureus* [6,35,51], Sendai virus [35,51], lactate dehydrogenase elevating virus [19], murine norovirus [15], *Pneumocystis spp.* [6], *Tritrichomonas spp.* [6], *Entamoeba spp.* [6] and fur mites [6,7,12,25,26]. EHM using PCR overcomes these limits by detecting small amounts of nucleic acids without the need for using infected sentinels for analysis, thereby reducing false-negative results and enabling detection even after pathogen shedding [5,22,34,49].

### Caging system and HM strategy

In Europe, EHM implementation largely depends on the type of cages and racks used. To the best of our knowledge, animal facilities in the German-speaking countries that predominantly use EHM for routine HM implement EDT. Luchins and co-authors found that in 2021 most facilities had implemented a hybrid (46%) or a SBS-based HM strategy (41%) while only a small number of facilities used EHM exclusively (11%) [23]. They reported that only 36% of the institutions were equipped exclusively with IVC racks with unfiltered air at the cage level, whereas 69% of the institutions were equipped with more than one caging system. In 2021, when evidence for SFSB was more limited, 40% of participants of that survey reported that caging and rack type were the most common barriers to the implementation of EHM. A combination of more than one cage type was also found in many facilities of our survey, with IVCs being the most common cage type (90% of the facilities) and open cages still present in 49% of the facilities. In our survey, 64% of all institutions still used predominantly animals for routine HM, whereas 20% used a hybrid HM strategy followed by 16% that used predominately EHM. Notably, more than half of the institutions equipped with at least 50% IVCs (59%) already used EHM to varying degrees (also in combination with animal testing).

Comparing our results with those of Luchins and co-authors [23] showed that, in German-speaking countries, the exclusive use of either EHM or animal-based HM was generally higher while the use of hybrid HM strategies was lower. Based on the answers from our survey, it was not possible to distinguish whether the IVCs had cage-level air filtration, which might have been a reason why EHM, specifically EDT, was not implemented. Furthermore, the questionnaire did not include further differentiation of the type of environmental samples used. Since SFSB is a more recent development, it is expected that in the future, more animal facilities will use this EHM method for routine HM because of its ease of implementation and, especially, because of its independence from the type of caging system used.

### The assessment of methodological advantages of EHM varies greatly depending on the experience level of participants

Microbiological diagnostics traditionally rely on methods like culture, serology, and microscopy. However, these are increasingly being replaced by molecular techniques, especially conventional and qPCR, which offer superior sensitivity and specificity [49,52,53]. PCR amplifies pathogen-specific DNA sequences using specific primers, enabling both qualitative and quantitative detection. This allows the detection of very small nucleic acid amounts, thereby reducing false-negative results and improving accuracy. These advantages are exploited by EHM. However, EHM is limited to identifying only those infectious agents included in the PCR panel. Therefore, combining EHM with necropsy, other microbiological methods, and pathological analyses, for example, for animals showing clinical signs or in gnotobiotic colonies where PCR alone may be insufficient, can improve detection.

The survey revealed that the participating institutions that already implemented EHM in their facility were well aware of these methodological advantages, which have been confirmed by numerous studies [5,9,20]. On the other hand, it must be pointed out that the diagnostic success strongly depends on the quality of the PCR assays design and nucleic acid isolated from the different sample matrices. Therefore, a comprehensive validation process for each PCR assay is essential to achieve maximum test accuracy and reduce the number of false-negative and false-positive test results. Nonetheless, participants currently using animals in this survey were concerned about false-positive test results for EHM. However, it is important to note that false-positives from PCR can also occur with samples from animals.

### Rack sanitation may impact EHM implementation

In our study, a lack of decontamination systems such as rack washers and autoclaves is an obstacle for participants of this survey that have not yet implemented EHM in their facility. As mentioned above, EDT and not SFSB is the predominant strategy for routine EHM in German-speaking countries. Therefore, effective decontamination measures should be in place to eliminate residual nucleic acids when EDT is used, especially when unexpected pathogens that are not accepted within a barrier are detected and subsequently eliminated through animal treatment or culling. This is particularly important for verifying therapeutic success by EHM samples and to exclude false-positive results in subsequent analyses. Positive PCR results do not provide information as to whether detected pathogens are infectious or not, but they do indicate the presence and shedding of the agent within the colony during exposure. Interpretation of such results is complicated by residual nucleic acids. Therefore, some facilities maintain lists of pathogens that are tolerated, particularly in experimental barriers, and only take action when pathogens outside these list are found or detected in “clean” units such as breeding barriers [5].

Decontamination of materials can be achieved by various strategies including washing, autoclaving, and gas decontamination with varying effectiveness. For example, *Corynebacterium bovis* DNA was eliminated by autoclaving racks [13,32]. Removal of nucleic acids from *Rodentibacter pneumotropicus, Helicobacter hepaticus*, murine norovirus or *Myocoptes musculinus* was achieved by washing racks followed autoclaving and cleaning/disinfecting the air handling unit [4,7,14,15]. Hydrogen peroxide-based decontamination of IVC racks with the air handling unit switched on successfully eradicated seven infectious agents after racks were sanitized at 180^o^F [18].

### Time efficacy of EHM is considered beneficial while costs are estimated equal or lower

The potential of EHM to generate time and cost savings is frequently debated in animal facilities in German-speaking countries. Among all participants, regardless of the different HM strategies used, time efficacy of EHM use was considered beneficial. This response is consistent with data from another survey that considers time savings as one of the most cited advantages of EHM [23]. While implementing EHM initially requires time for employee training, the time required for set-up, collection, and submission of environmental samples to the diagnostic laboratory is rather low. In contrast, using sentinels requires more time due to animal care, weekly soiled bedding collection during cage changes, and preparation of animals for shipping [41].

Notably, cost concerns persist among participants that have not yet adopted EHM, despite existing literature demonstrating measurable cost reductions. Regarding costs, a more divergent picture emerges among the participants. Animal facilities that switched from sentinels to predominantly EHM reported that they were able to reduce costs. Similarly, the survey of Luchins and co-authors [23] showed that the participating facilities view the cost factor as an advantage when using environmental samples. Costs depend on whether EHM is used as a substitute for sentinel animal testing, either as a complete replacement method or alternately with animal testing or whether parallel testing is carried out, which incurs additional costs. Furthermore, the initial implementation of EHM in a facility incurs set-up costs, in addition to operational costs for sampling material and diagnostics. On the contrary, the use of sentinels often results in increased costs for HM due to the purchase of sentinel animals, if necessary, their housing and care, shipment to a diagnostic laboratory, in addition to the diagnostic costs.

According to Luchins et al., switching from sentinels to EHM resulted in a cost saving of 26% over two years [41]. However, if previous testing relied predominantly on serological analysis, the switch to molecular testing for EHM sample analyses can make monitoring more expensive [23]. One efficient strategy to reduce costs efficiently is the pooling of samples. A previous study has shown that even pooled EHM samples from up to 10 air handling units were superior to sentinel analysis [5]. Prevalence studies have demonstrated that the dilution effect may be negligible to a certain extent, depending on the respective pathogen and its excretion [4,13,14,38].

In general, it is advisable to collect duplicate samples from each rack or filter unit to ensure that backup samples are available for further analysis. Unexpected positive results, that is, usually the detection of infectious agents whose presence in the colony was previously unknown, should be confirmed and followed up systematically by re-testing backup samples. Re-testing of individual backup samples does not only allow epidemiological investigations quickly, but also saves money, as testing for individual microorganisms is much cheaper than testing an entire panel of infectious agents [5]. To confirm unexpected positive PCR results and exclude potential false-positive findings caused by residual nucleic acids in the rack, it may be necessary to test individual colony animals using alternative testing methods.

### Further training may increase EHM implementation in facilities

In our survey, the questionnaire was answered by primarily the managers of the animal facility and/or veterinarians as they are typically responsible for the decision-making processes in European animal facilities. These roles are often closely interlinked in both strategic planning and operational oversight. Questions regarding the self-assessment of participants resulted in an overall medium to high knowledge about HM in general. In contrast, the specific, self-assessed knowledge about EHM was considerably lower compared to animal-based HM strategies. In line with these answers, the majority of participants who currently predominantly use animals for HM stated that they might implement EHM if they could attend further training for its use. This suggests that detailed information on the use of environmental samples for HM purposes, with all advantages and disadvantages, has not yet reached all those responsible for determining an institution’s HM strategy. Appropriate training, scientific talks, workshops, and further literature about EHM are therefore essential to improve knowledge transfer. In addition, the access to standard operating procedures with detailed protocols for EHM sampling techniques may be beneficial. The Committee for Hygiene of the GV-SOLAS and the FELASA have been supporting awareness and training not only on the topic of HM but also on EHM.

### New recommendations may increase EHM implementation in facilities

While the current FELASA recommendations on HM in laboratory rodent facilities relate primarily to the examination of animals and/or sentinel animals, the analysis of environmental samples is only briefly mentioned [1]. The survey revealed that revised recommendations on HM in laboratory rodent facilities from a source such as the FELASA would most likely influence the choice of the HM strategy. More than half of the participants indicated that they would definitely or likely implement EHM if the methodology were to be recommended by the FELASA. In this regard, a new FELASA recommendation for the methodology for HM of mice maintained in IVCs including more details concerning EHM is expected to be published soon.

Nevertheless, most participants who currently predominantly use animals for HM in this survey would rather implement a hybrid method than exclusively rely on EHM. This is plausible and also reflects the authors’ own experiences. Most animal facilities that have not yet had experience with EHM generally test both animals and environmental samples in parallel over a certain period of time in order to gain experiences and to test the integration of environmental sampling in their own HM set-up.

Despite the reservations, the general acceptance of health certificates based on EHM by institutions of this survey was high. Six participants who implement EHM did not accept animal imports from other facilities with health certificates based on EHM results. The reason for this rejection cannot be elucidated from the responses. A plausible and more probable explanation could be that imports from other institutions are generally not allowed in these animal facilities. A study describing the implementation of EHM in their facility showed that over the course of three years of using EHM, more than 300 animal shipments were made to 135 other institutions in 14 countries. None of the receiving institutions reported the detection of excluded pathogens in any of these shipments [20].

### EHM implementation reduces the number of animals used and contributes to the 3Rs

The use of EHM for routine HM can make a significant contribution to the 3Rs [42] by replacement or at least a reduction of animals, particularly SBS, thereby significantly reducing the number of animals required. Typically, one sentinel cage is used per rack, so thousands of animals are used each year in research institutions solely for routine HM [22]. Our survey revealed a reduction of 6,331 animals in facilities that have already switched to EHM or adopted a hybrid monitoring strategy. Additionally, another large proportion of facilities that have not yet implemented EHM anticipated a similar reduction of animals in the future. The extent of reduction varied considerably across facilities, which can be attributed to differences in the size of the animal facilities and the degree of implementation. Some facilities switched completely to EHM while others continued to use sentinels and environmental sampling in parallel in a hybrid approach. Several publications comparing the use of sentinels and EHM also report a significant saving potential of several thousand animals per year [5,20,41]. In a survey conducted by Luchins and co-authors, participating institutions reported that the use of EHM had led to a reduction of over 20,000 animals per year [23]. The authors of a recently published systematic review conclude that there is a scientific and ethical necessity to revise HM programs and replace sentinels with EHM [22].

### Limitations of this study

Despite the valuable insights gained, this study has several limitations that should be considered when interpreting the results. The use of self-reported data may have introduced response bias, particularly in relation to perceived barriers such as time, cost, and the need for decontamination. Although the variability in participants’ EHM knowledge could have affected cost and time estimates, this diversity reflects ‘real-world’ opinions rather than those of an idealized expert subset. Moreover, the questionnaire did not differentiate between types of IVC systems (cage- versus rack-level filtration) or environmental sampling methods, potentially obscuring technical challenges relevant to EHM implementation. To enhance future research, more detailed questionnaires, broader participant inclusion, and collection of technical data are recommended to support a more comprehensive understanding of EHM adoption across facilities in German-speaking countries.

## Conclusion

Although the majority of institutions in German-speaking countries, which are equipped with IVCs, already use EHM to varying extents, our survey showed that most laboratory animal facilities still predominantly use SBS for their HM programs, which are usually killed for microbiological examination. There are still reservations against PCR-based EHM, particularly regarding costs, its reliability, and the number of false-positive results. Likewise, the lack of knowledge and suitable equipment for rack decontamination are major obstacles to the implementation of EHM. The inclusion of EHM in the FELASA recommendations and the provision of training and further education for those responsible for HM will most likely contribute to a better acceptance of EHM in German-speaking countries in the future.

In conclusion, numerous comparative studies almost unanimously demonstrate that the use of EHM is superior to SBS, enabling the detection of pathogens that are not or not reliably transmitted through soiled bedding. The implementation of EHM holds significant potential to reduce or replace laboratory animals used worldwide for the purpose of HM while improving HM validity by increased diagnostic sensitivity.

## Supporting information

S1 Table Structure of the questionnaire on health monitoring containing 5 sections divided into 33 questions with potential answers and 1 comment field. (DOCX)
