# Supplementary material for: A survey on the implementation of environmental health monitoring in mouse facilities in German-speaking countries
Source: PLoS One. 2025 Oct 23;20(10):e0334442. doi: 10.1371/journal.pone.0334442 (PMC12548899; doi:10.1371/journal.pone.0334442)
Supplement: S1 Table — (DOCX) [file pone.0334442.s001.docx]

Supporting Information

**S1 Table: Structure of the questionnaire on health monitoring containing 5 sections divided into 33 questions with potential answers and 1 comment field**

| Section | Question Number | Wording | Options |
| --- | --- | --- | --- |
| **1 - Facility and Husbandry** | 1 | Location of facility | Germany/Austria/ Switzerland |
|  | 2 | Type of facility | Academic/Industry |
|  | 3a | Person responsible for health monitoring: veterinarian | Yes/No |
|  | 3b | Person responsible for health monitoring: biologist | Yes/No |
|  | 3c | Person responsible for health monitoring: facility manager | Yes/No |
|  | 3d | Person responsible for health monitoring: other | Yes/No |
|  | 4 | Total number of cages | less than 1,000/1,000-10,000/more than10,000 |
|  | 5a | Ratio of open cages to the total number of cages | Percentage |
|  | 5b | Ratio of IVCs without filtered air at cage level to the total number of cages | Percentage |
|  | 5c | Ratio of IVCs with filtered air at cage level to the total number of cages | Percentage |
|  | 5d | Ratio of other cages to the total number of cages | Percentage |
| **2 - Level of Knowledge** | 6 | Level of knowledge in the facility about health monitoring in mice in general | Very high/high/medium/ low/very low |
|  | 7 | Level of knowledge in the facility about health monitoring with animals (soiled bedding sentinels, contact sentinels, colony animals) | Very high/high/medium/ low/very low/ |
|  | 8 | Level of knowledge in the facility about health monitoring with environmental samples from IVC systems (environmental health monitoring) | Very high/high/medium/ low/very low/ |
|  | 9a | Which sources do you use regularly to increase your knowledge about health monitoring?: commercial diagnostic laboratories | Yes/no |
|  | 9b | Which sources do you use regularly to increase your knowledge about health monitoring?: scientific literature | Yes/no |
|  | 9c | Which sources do you use regularly to increase your knowledge about health monitoring?: scientific meetings | Yes/no |
|  | 9d | Which sources do you use regularly to increase your knowledge about health monitoring?: colleagues | Yes/no |
|  | 9e | Which sources do you use regularly to increase your knowledge about health monitoring?: none of the above-mentioned | Yes/no |
| **3 - Health Monitoring** | 10 | Do you have IVCs (without filtered air at cage level) and do you use environmental health monitoring? | Yes/no/we have no IVC cages without filtered air at cage level |
|  | 11 | Do you have other cages besides IVCs without filtered air at cage level and do you use environmental health monitoring? | Yes/no/we have no other cages besides IVCs without filtered air at cage level |
|  | 12 | Which health monitoring strategy do you use predominantly? | Environmental health monitoring/ animals (soiled bedding sentinels, contact sentinels, colony animals) / combination of environmental health monitoring and animals (hybrid) |
|  | 13a | Which methods are used to test your sentinels or colony animals?: PCR | Yes/no |
|  | 13b | Which methods are used to test your sentinels or colony animals?: serology | Yes/no |
|  | 13c | Which methods are used to test your sentinels or colony animals?: parasitology | Yes/no |
|  | 13d | Which methods are used to test your sentinels or colony animals?: microbiology | Yes/no |
|  | 13e | Which methods are used to test your sentinels or colony animals?: pathology | Yes/no |
|  | 13f | Which methods are used to test your sentinels or colony animals?: we do not test soiled bedding sentinels, contact sentinels or colony animals for routine health monitoring | Yes/no |
|  | 14 | Who is predominantly conducting the diagnostics for your routine health monitoring? | In-house/external laboratories |
|  | 15a | Which other diagnostic methods do you use in addition to your routine health monitoring with animals and/or environmental samples?: examination of animals e.g. sentinels and colony animals if predominantly environmental samples are analysed | Yes/no |
|  | 15b | Which other diagnostic methods do you use in addition to your routine health monitoring with animals and/or environmental samples?: testing of sick/symptomatic/dead animals | Yes/no |
|  | 15c | Which other diagnostic methods do you use in addition to your routine health monitoring with animals and/or environmental samples?: environmental samples from cages (e.g. swabs) | Yes/no |
|  | 15d | Which other diagnostic methods do you use in addition to your routine health monitoring with animals and/or environmental samples?: no additional diagnostic method | Yes/no |
| **4 - Decontamination** | 16 | Does your facility have the possibility to wash cage racks? | For all racks/partially)/no |
|  | 17 | Does your facility have the possibility to autoclave cage racks? | For all racks/partially)/no |
| **5 - Comparison of environmental health monitoring and soiled bedding sentinel** | 18a | Reduction of the use of animals: how many animals do you save by using environmental health monitoring per year? (do not answer if you do not use environmental health monitoring) | Absolute number |
|  | 18b | Reduction of the use of animals: how many animals do you save by using environmental health monitoring per year as a percentage of all animals used for health monitoring? (do not answer if you do not use environmental health monitoring) | Percentage |
|  | 19a | Reduction of the use of animals: how many animals would you save per year if you use environmental health monitoring? (do not answer if you use environmental health monitoring) | Absolute number |
|  | 19b | Reduction of the use of animals: how many animals would you save per year if you use environmental health monitoring as a percentage of all animals used? (do not answer if you use environmental health monitoring) | Percentage |
|  | 20 | Costs compared to those when using soiled bedding sentinels: how do you estimate the costs if you are using environmental health monitoring or will switch to environmental health monitoring in the future? | Higher/equal/lower |
|  | 21 | Time needed compared to that when using soiled bedding sentinels: how do you estimate the time needed if you are using environmental health monitoring or will switch to environmental health monitoring in the future? | Higher/equal/lower |
|  | 22 | Reliability of results: how do you estimate the reliability of results from environmental health monitoring compared to those using soiled bedding sentinels? | Higher/equal/lower |
|  | 23 | Sensitivity of results: how do you estimate the sensitivity of results from environmental health monitoring compared to that using soiled bedding sentinels? | Higher/equal/lower |
|  | 24 | False-negative results: how do you estimate the number of false-negative results from environmental health monitoring compared to that using soiled bedding sentinels? | Higher/equal/lower |
|  | 25 | False-positive results: how do you estimate the number of false-positive results from environmental health monitoring compared to that using soiled bedding sentinels? | Higher/equal/lower |
|  | 26 | If you currently have an IVC system with filtered air at the cage level: would you use environmental health monitoring if you could use suitable IVC systems without filtered air at the cage level? | Yes/no/we do not use an IVC system with filtered air at the cage level |
|  | 27 | If you currently do not have the means to decontaminate IVCs: would you implement environmental health monitoring if you could decontaminate IVCs in your facility? | Yes/no/we can decontaminate IVCs |
|  | 28 | How high is the probability of conducting routine health monitoring in your facility predominantly by environmental health monitoring within the next two years? | very high/likely/medium/ unlikely/not at all/we already use predominantly environmental health monitoring in our facility |
|  | 29 | How high is the probability of using a hybrid system (combination of environmental health monitoring and animal examination: sentinels and/or colony animals) for routine health monitoring in your facility within the next two years? | very high/likely/medium/ unlikely/not at all/we already use predominantly a hybrid system in our facility |
|  | 30 | Does your facility accept animals from other facilities whose health certificates are based completely or partially on environmental health monitoring results? | Yes/no |
|  | 31 | Are your health certificates, which are based completely or partially on environmental health monitoring results, accepted when you export animals to other facilities? | very high/mostly/medium/ low/not at all/we do not use environmental health monitoring |
|  | 32 | Would you use environmental health monitoring if you are provided suitable further training on environmental health monitoring? | Yes/likely/maybe/unlikely/ no/we already use environmental health monitoring |
|  | 33 | Would you use environmental health monitoring if this method is part of the FELASA recommendations? | Yes/likely/maybe/unlikely/ no/we already use environmental health monitoring independent of the FELASA recommendations |
|  | 34 | Comments of participants |  |

Question type: Single answer, Multiple answers, Open-Ended (Freestyle-Text)
